# Emergency Department Clinicians’ Attitudes Toward Opioid Use Disorder and Emergency Department-initiated Buprenorphine Treatment: A Mixed-Methods Study

**DOI:** 10.5811/westjem.2019.11.44382

**Published:** 2020-02-21

**Authors:** Dana D. Im, Anita Chary, Anna L. Condella, Hurnan Vongsachang, Lucas C. Carlson, Lara Vogel, Alister Martin, Nathan Kunzler, Scott G. Weiner, Margaret Samuels-Kalow

**Affiliations:** * Massachusetts General Hospital, Department of Emergency Medicine, Boston, Massachusetts; † Brigham and Women’s Hospital, Department of Emergency Medicine, Boston, Massachusetts; ‡ University of Southern California, Keck School of Medicine, Department of Emergency Medicine, Los Angeles, California

## Abstract

**Introduction:**

Emergency department (ED) visits related to opioid use disorder (OUD) have increased nearly twofold over the last decade. Treatment with buprenorphine has been demonstrated to decrease opioid-related overdose deaths. In this study, we aimed to better understand ED clinicians’ attitudes toward the initiation of buprenorphine treatment in the ED.

**Methods:**

We performed a mixed-methods study consisting of a survey of 174 ED clinicians (attending physicians, residents, and physician assistants) and semi-structured interviews with 17 attending emergency physicians at a tertiary-care academic hospital.

**Results:**

A total of 93 ED clinicians (53% of those contacted) completed the survey. While 80% of respondents agreed that buprenorphine should be administered in the ED for patients requesting treatment, only 44% felt that they were prepared to discuss medication for addiction treatment. Compared to clinicians with fewer than five years of practice, those with greater experience were less likely to approve of ED-initiated buprenorphine. In our qualitative analysis, physicians had differing perspectives on the role that the ED should play in treating OUD. Most physicians felt that a buprenorphine-based intervention in the ED would be feasible with institutional support, including training opportunities, protocol support within the electronic health record, counseling and support staff, and a robust referral system for outpatient follow-up.

**Conclusion:**

ED clinicians’ perception of buprenorphine varied by years of practice and training level. Most ED clinicians did not feel prepared to initiate buprenorphine in the ED. Qualitative interviews identified several addressable barriers to ED-initiated buprenorphine.

## INTRODUCTION

### Background

Emergency department (ED) visits related to opioid use disorder (OUD) have increased nearly twofold over the last decade.^1^ As a critical access point for patients with OUD, the ED is well positioned to provide and link patients to OUD treatment.^2^ However, current practice in United States EDs for patients seeking treatment for OUD is referral to addiction treatment services, which often consist of abstinence-based programs or psychosocial interventions.^3^

Buprenorphine is a first-line medication for addiction treatment (MAT) of OUD.^4^ Treatment with buprenorphine decreases non-medical opioid use and opioid-related overdose deaths while improving retention in treatment compared to patients receiving abstinence-based treatment or psychosocial intervention.^5^–^8^ A recent randomized controlled study demonstrated that when buprenorphine treatment was initiated in the ED, patients were more likely to remain engaged in treatment compared to brief intervention and referral for treatment.^9^ ED-initiated buprenorphine was also found to be cost-effective compared with referral to community-based treatment or combined brief intervention and referral.^10^

### Importance

Several EDs have launched ED-initiated treatment programs with buprenorphine.^2^,^11^–^14^ Legislative changes are also underway to incorporate initiation of buprenorphine into ED management of patients of OUD. For example, a new State of Massachusetts law requires acute care hospitals that provide emergency services to have the capacity to initiate opioid agonist therapy to patients after an opioid-related overdose, and to directly connect the patients to continuing treatment prior to discharge.^15^ Despite the growing national momentum toward offering buprenorphine in the ED, little is known about ED clinicians’ attitudes related to this practice.^16^,^17^ To work toward the goal of improving care for patients with OUD in the ED, it will be important to better understand clinicians’ diverse views on and perceived barriers to the practice of initiating buprenorphine in the ED.

### Goals of This Investigation

The objective of this study was to better understand ED clinicians’ perceptions of OUD and ED-initiated buprenorphine treatment. This was a mixed-methods study consisting of a survey and in-depth qualitative interviews of ED clinicians. The survey phase aimed to understand ED clinicians’ perceptions of ED-initiated buprenorphine treatment, in addition to their attitudes, clinical practice, and self-perceived preparedness related to caring for patients with OUD. The goal of the qualitative interview phase was to explore emergency physicians’ perceptions about their current management options for OUD, characterize their opinions about ED-initiated buprenorphine, and identify addressable barriers to prescribing buprenorphine in the ED. While the survey phase focused on measuring how many ED clinicians have certain perceptions, the purposive, in-depth qualitative interviews were designed to uncover a range of opinions and to identify new ideas and concepts, embedded in real-life experiences that frame OUD treatment for emergency physicians.

**Population Health Research Capsule**

What do we already know about this issue?

Treatment of opioid use disorder (OUD) with buprenorphine has been shown to decrease opioid-related overdose deaths while improving retention in treatment.

What was the research question?

How do emergency department (ED) clinicians perceive opioid use disorder and ED-initiated buprenorphine?

What was the major finding of the study?

Most ED clinicians supported ED-initiation of buprenorphine, which would be feasible with robust institutional support.

How does this improve population health?

This study provides potential solutions to facilitate the initiation of buprenorphine in the ED and transform the delivery of emergency care for OUD patients.

## METHODS

We conducted a cross-sectional survey of ED clinicians (attending physicians, resident physicians, and physician assistants) and individual semi-structured interviews with emergency medicine (EM) attending physicians working in an ED at a tertiary-care academic hospital with an annual volume of 65,000 patients. The study was approved by the study site’s institutional review board.

### Survey

The sampling frame for the survey phase consisted of attending physicians, residents, and physician assistants (n = 174) from December 2017 to February 2018. A request to participate along with a link to the de-identified survey was emailed to ED clinicians. The survey was administered via Qualtrics (Qualtrics, Provo, Utah). ED clinicians received an initial request and two reminder emails and an incentive of a $10 gift card for survey completion. As an exploratory study, a sample size calculation was not performed a priori, but rather investigators aimed for a response rate of >50% with a goal of recruiting approximately 100 participants.

A previously studied survey instrument designed for internal medicine physicians was adapted to assess ED clinicians’ attitudes, exposure, clinical practice, and preparedness related to caring for patients with OUD on an 11-point Likert scale.^18^ Two questions were specific to understanding perceptions of buprenorphine treatment and whether it should be initiated in the ED. Participants’ role and their total years of practice in EM after graduation from medical or physician assistant school were also recorded.

We selected our outcomes a priori and performed the Kruskal-Wallis with Bonferroni adjusted pairwise Mann-Whitney tests to determine the differences in the responses based on years of practice and roles. Seven participants with missing responses were removed from data analysis. We used Stata version 13.1 (Stata Corporation, College Station, TX) for all statistical analyses.

### Qualitative Interview

For the interview phase of the study, we contacted all ED attending physicians (n = 72 by email), informed them of the study, and invited them to be interviewed on a voluntary basis in February–March, 2018. Participants were offered a $50 gift card. Study participants were recruited until thematic saturation was reached, which is the point at which no new themes emerged. We recruited 17 attending physicians to interviews, in line with typical sample size employed to achieve thematic saturation in qualitative studies.^19^

Qualitative, semi-structured interviews were conducted individually in person by a research assistant (H.V.) trained in in-depth interviewing by an expert in qualitative methodology (A.C.). Informed consent was verbally obtained before each interview. Interview questions focused on emergency physicians’ experiences treating patients with OUD as well as attitudes towards buprenorphine initiation in the ED (Table 1). Basic demographic information was collected about each participant’s number of years of practice, average number of shifts worked per month, and fellowship training.

All interviews were recorded, de-identified and professionally transcribed. Investigators developed a codebook based on preliminary review of six transcripts. Subsequently, individual interviews were coded independently by four investigators (D.I., A.C., L.V., and L.C.), with NVivo version 12 (QSR International, Melbourne, Australia). D.I., A.C., and H.V. serially reviewed coded transcripts and discussed discrepancies until reaching consensus. Themes were identified using a modified grounded theory approach, and thematic saturation was determined by team consensus.

## RESULTS

### Survey Results

Of the 100 survey respondents, 93 had complete responses (57% response rate, 53% completion rate) and were included for analysis. Table 2 summarizes the characteristics of survey respondents. Of the respondents surveyed, 88% agreed that buprenorphine should be administered in the ED for patients requesting treatment for OUD. However, only 44% of ED clinicians reported that they felt prepared to discuss MAT options with patients. Table 3 summarizes ED clinicians’ attitudes related to OUD and buprenorphine by years of practice and roles in the ED. Compared to clinicians with more than five years of practice, those with fewer years of practice were (1) more likely to believe that OUD is like other chronic diseases; (2) more likely to approve of ED-initiated buprenorphine; and (3) less likely to believe that buprenorphine replaces one addiction with another (p<0.01 for each). Compared to attending physicians, residents were less likely to believe that OUD is different from other chronic diseases (p<0.03).

Attending physicians and residents viewed ED-initiated buprenorphine more favorably than physician assistants (p<0.01). Compared to physician assistants, residents were also less likely to view buprenorphine as replacing one addiction with another (p<0.01). Compared to clinicians with fewer than five years of practice, those with more years of practice were more likely to feel prepared to discuss overdose prevention and naloxone with patients (p<0.03) (Table 4). Attending physicians were more likely to feel prepared to discuss harm reduction with patients than residents (p=0.01).

### Qualitative Interview Results

Table 5 summarizes the characteristics of the 17 interviewees. Several themes emerged regarding the following: (1) emergency physicians’ views of current ED practices to manage OUD; (2) perceptions of ED induction of buprenorphine for OUD treatment; (3) clinician-level barriers and solutions to initiating buprenorphine in the ED; and (4) systems-level barriers and solutions to initiating buprenorphine in the ED.

### Current ED-based Approaches to Manage Patients Seeking Treatment for OUD

The majority of the emergency physicians (EPs) described current practices as consulting social work (if available) and providing a list of detoxification facilities to patients. As one participant observed, “My practice has been pretty much what emergency physicians have largely done, which is I give them the badly photocopied list of treatment options and let them walk out the door.”

EPs expressed frustration, anger, helplessness, sadness, and dissatisfaction when describing their current practices to manage patients seeking treatment for OUD in the ED setting. Their emotions stemmed from the inadequate and limited nature of the current management options. One emergency physician summarized: “It’s really frustrating, and I feel kind of helpless sometimes – where we’re doing the bare minimum to get them discharged, and that’s kind of the best we can do, and the rest of it’s on them.” Many of the participants associated their dissatisfaction with the sentiment of “temporizing things without feeling like we’re actually making a meaningful difference” in patients’ lives.

### Emergency Physicians’ Views of ED-initiated Buprenorphine Treatment

EPs expressed their view of buprenorphine as an effective treatment option for OUD. As one participant elaborated, “I’ve heard that when well managed and when well coordinated, that it has a whole lot better efficacy than some of the other things that we have seen, certainly compared to the non-medication-assisted therapies.”

Despite favorable views of buprenorphine in general, only a minority of the interviewees were in favor of ED-initiated buprenorphine. Those who supported ED-initiated buprenorphine often cited the duty of EM as a medical specialty to improve public health. One EP described EM as an all-encompassing specialty, with the ED serving as a point of capture for underserved populations: “When [patients] are [in the ED] for whatever issue, whether it be an overdose or some other medical process, it’d be a great way to capture them and put them into some sort of system at least to get [the treatment] started.”

Reluctance to support ED-based buprenorphine treatment stemmed from three major concerns. First, interviewees viewed prescribing buprenorphine as not within the scope of EM practice. One participant described the current ED practice of deferring long-term management of chronic illnesses to outpatient clinicians, and applied this to using buprenorphine to treat OUD: “My impression is that it’s not necessarily a great thing for emergency physicians to be primarily involved with those patients because – just like I don’t manage people’s diabetes long term and I don’t manage their blood pressure long term, I don’t see long-term management of the buprenorphine as within our wheelhouse.”

A second concern about prescribing buprenorphine related to patients’ potential misuse of the medication. Interview participants expressed belief that buprenorphine is a highly diverted medication, which would encourage patients to either abuse or sell ED-prescribed buprenorphine. One physician stated, “What I do fear is that there is a potential for emergency medicine to be seen as like a way to potentiate kind of bad habits if people know like ‘oh, if I go and I ask for buprenorphine, I’ll get a script for it and I can somehow misuse that.’ That’s one of my concerns. I know buprenorphine has some kind of misuse prevention kind of built into the way it’s formulated, but I still think it’s sold on the street and has a street value and is – it could be misused. I just want to be careful that I’m not adding to the problem and that I really am alleviating the problem by me participating in this way.”

Third, physicians vocalized their concerns about inadvertently harming patients with buprenorphine. They expressed reluctance to start prescribing a new medication that could result in overdose or co-ingestion with other sedatives, such as benzodiazepines. Another physician offered, “If they take higher than normal doses to get an effect and you end up causing a death or an inadvertent overdose because of the way it’s done and the mechanism of action and somebody wants to, you end up doing more harm.”

### Clinician-level Barriers and Solutions to Provision of MAT in the ED

EPs also identified three major clinician-level addressable barriers potential and solutions. First, EPs commonly cited that the current length of the waiver training is burdensome. Under the Drug Addiction Treatment Act of 2000 (DATA 2000), physicians are required to complete an eight-hour training to qualify for a waiver to prescribe and dispense buprenorphine.^20^ As a potential solution, participants suggested providing financial or academic incentives for completing the waiver training. In addition to the waiver training required for potential prescribers, an institution-wide educational campaign was recommended for other stakeholders in the ED, including nurses, non-physician clinicians, administrative staff, and other support staff. As one participant stated, “I just think there would have to be an emergency department-wide educational process. The nurses need to be on board. The whole team needs to be on board.”

A second barrier noted was the time-consuming nature of building therapeutic relationships in order to identify ideal candidates for buprenorphine treatment and to engage these patients for buprenorphine induction in the ED. As one EP expressed, “It’s unrealistic for the ER doc to do that because it takes time.” Another commented about resource utilization: “The reality is it’s time away from other patients.” One participant made an analogy to providing medical forensic care to victims of sexual assault or abuse, stating that it is a skillset she has acquired in her training, but has not used frequently enough to feel confident in her ability to conduct an exam efficiently, effectively, and safely. She advocated that just as specialized practitioners, such as a sexual assault nurse examiner, are better equipped with training, practice, and time to conduct an exam for forensic evidence collection, EDs should employ dedicated, specialized staff (social worker, advocate, or addiction specialist) to identify patients ideal for ED-initiation of buprenorphine, to discuss instructions on how to start buprenorphine, and to ensure outpatient follow-up.

A third clinician-level barrier identified was a reported lack of motivation to start patients on buprenorphine in the ED because of delayed clinical gratification. Participants expressed frustration with the inability to see the impact of engaging patients to start MAT in the ED on long-term opiate use. One potential solution offered was to create a mechanism that tracks patients’ engagement in outpatient MAT after ED discharge and reports it back to ED prescribers. As one participant stated, “[it] would be really key to be able to show that this was having positive outcomes for people and I think that kind of positive feedback would be really helpful.” See Table 6 for additional supporting quotes.

### System-level Barriers and Solutions to Provision of MAT in the ED

Interviewees described three major systems-level barriers and solutions to offering buprenorphine in the ED and their potential solutions. First, EPs expressed discomfort with prescribing buprenorphine in the ED without the ability to ensure outpatient follow-up. In describing the need for establishing a long-term plan for patients being considered for buprenorphine, interviewees identified the anticipated gaps in the outpatient follow-up system. One EP questioned, “Like, what if the person can’t [see] the PCP for 20 days? Then, all of a sudden, you’re the one prescribing 20 days of [buprenorphine/naloxone], which – I don’t know – I might feel uncomfortable doing that with that patient population. So, I think it would have to be some sort of strict process of like, we’ll give you two doses or something like that, and then [connect them to] a good follow-up system to go to somebody who’s going to do it long term. Because I think that’s the issue with a lot of ED clinicians is we’re not going to be the ones following them.”

As a potential solution, EPs looked to the system-level approach of using electronic health record (EHR) integration for providing cohesive addiction treatment. EHR integration can enhance the ability to place electronic orders for referrals and to track patients using mechanisms such as a prescription drug monitoring program. Individualized care plans, which many EHRs have integrated for complex care patients, were also recommended to guide ED management of patients with OUD.

Participants raised a second systems-level barrier of possible financial barriers for patients to continue on buprenorphine after ED-induction. Interviewees suggested that variability in insurance coverage may prohibit patients from continuing on buprenorphine after induction in the ED. In addition to having dedicated ED staff helping patients navigate the healthcare system and ensuring follow-up, physicians suggested providing ready-to-go buprenorphine in supply kits (3–7 days) or in a depot form. Physicians suggested this would ensure that patients would have the needed supply until they can be seen by an outpatient clinician and potentially minimize diversion risks.

A third system-level barrier was the anticipated increase in ED volume related to patients requesting OUD treatment. Some physicians expressed concerns about MAT workload being shifted to EPs from outpatient clinicians. While some participants worried about the potential strain on the ED, others were optimistic about an eventual decrease in the number of ED visits related to overdoses and injuries associated with OUD. Physicians suggested a potential solution for reducing the burden on EPs was to institutionalize clear clinical protocols for initiating buprenorphine in the ED. Clinical protocols similar to those that exist for risk stratifying and managing patients with chest pain could be developed for initiation of buprenorphine in the ED for patients with OUD. Additional quotes regarding these themes are available in Table 7.

## DISCUSSION

Our mixed-methods approach allowed a nuanced analysis of ED clinicians’ attitudes toward OUD and ED-initiation of buprenorphine for OUD treatment. Recent evidence suggests that ED attending physicians and residents view patients with substance use disorders differently than those with other medical conditions.^17^ Similarly, our data showed that some ED clinicians viewed OUD as different from other chronic disease, but this group represented only a minority of our surveyed ED clinicians (34% of surveyed attending physicians, residents, and physician assistants). Interestingly, our in-depth qualitative interviews with attending physicians revealed nuances in the negative emotions such as helplessness, sadness, and frustration associated with OUD and the currently limited ED-based treatment options for OUD. These feelings were directed at clinicians’ own inability to effectively help patients with OUD, and did not seem to be directed at the patient population itself. Unlike other studies that have shown health professionals’ general negative attitudes toward patients with OUD, we differentiate clinicians’ frustrations at the status quo from their negative feelings toward working with this patient population.^16^,^17^,^22^,^23^ These data can inform development of future initiatives to redesign care for patients requesting treatment for OUD.

Our analysis of the survey results captured another nuance in ED clinicians’ attitudes toward OUD and buprenorphine: the minority of ED clinicians who negatively viewed OUD and ED-initiated buprenorphine had a disproportionate representation of clinicians with more experience. Similar patterns existed in the attitudes toward buprenorphine and its administration in the ED among survey respondents. The difference in the attitudes toward OUD and buprenorphine by years of practice may reflect changing attitudes toward OUD and a higher number of negative experiences associated with providing treatment for patients with OUD over time. We also attribute this difference to the increased education about OUD for more recently trained ED clinicians with the increased public awareness of the opioid epidemic, particularly regarding the second and third waves of rapid rise in overdose deaths in 2010 and 2013, involving heroin and synthetic opioids, respectively.^24^,^25^

We also found a difference in the attitudes toward ED-initiated buprenorphine between EPs (attendings and residents) and physician assistants. Institution-wide initiatives for ED-initiation of buprenorphine must take into account the important roles that non-physician clinicians can play, with physician assistants and nurse practitioners also qualified to prescribe buprenorphine after obtaining a waiver. Notably, the Comprehensive Addiction and Recovery Act signed into law in 2016 requires qualifying non-physician clinicians to complete 24 hours of training to be eligible for a waiver, compared to eight hours for physicians.^26^

While 80% of our survey respondents supported ED-initiation of buprenorphine, less than half expressed comfort even with discussing MAT options with patients, let alone initiating MAT in the ED. Our findings regarding clinicians’ preparedness are similar to the results of a recent survey study: a minority of EPs felt prepared to connect patients with OUD to outpatient care or to initiate buprenorphine.^27^ Our qualitative interviews further elucidated why ED clinicians feel underprepared and reluctant to treat patients with buprenorphine in the ED. Our interviews confirmed that EPs were reluctant to initiate buprenorphine despite their understanding of the scientific evidence behind the effectiveness of buprenorphine. Their reluctance originated from presumed unintended consequences, including diversion, abuse, and accidental overdoses. These concerns represent a double standard applied to buprenorphine, compared to other dangerous medications commonly prescribed by EPs without any special training or waivers. To dispel these concerns, we recommend describing the required Drug Enforcement Administration waiver course as a tool that empowers EPs with new knowledge and skillsets to transform addiction care—akin to mastering techniques for nerve blocks and difficulty airways.

Our preliminary study identified a mix of barriers to ED-initiated buprenorphine at the clinician and system levels, but all the suggested solutions were beyond what one clinician could do, highlighting the need for institutional support. EPs supported integrated healthcare delivery systems that seamlessly coordinate care between the ED and outpatient providers with central databases that allow creation and visualization of complex care plans and prior prescriptions. As managed care becomes increasingly pervasive in healthcare for both privately and publicly insured individuals, we anticipate more healthcare organizations will be incentivized to implement initiatives that coordinate care for chronic, complex conditions such as OUD. Managed care organizations aligning payment incentives with performance goals present opportunities for EM as a specialty to advocate for integrating care to support ED-initiated buprenorphine programs. Building on this preliminary study results, future research can include a larger sample from EDs across the spectrum (academic, community, urban, and rural) to gather more generalizable results. Future directions also include implementation of our suggested clinician- and systems-level solutions and analysis of the impact of the interventions on initiating buprenorphine in the ED.

## LIMITATIONS

Our study is limited by the small sample size, which may influence the generalizability of the results. Second, our survey study had a low response rate (53% completion rate), which may have contributed to sampling bias. Those who responded to survey and interview invitations may have chosen to participate due to interests in OUD not present in the general population of participating physicians. Third, our study took place in Massachusetts, which is one of the states most affected by the opioid epidemic.^21^ Clinicians in this practice setting have significant exposure to MAT strategies, and acceptability of buprenorphine may reflect regional emphasis on this issue. At the time of the study, another hospital in our health system initiated a program to encourage ED attending physicians to become waivered to prescribe buprenorphine. Our study participants’ exposure to this program within the same health system may have affected the external validity of our findings. The geographic limits may also impact the generalizability of the results.

Fourth, our study relied on clinicians whose primary appointment is at an ED at a tertiary-care academic hospital, which is more likely to be equipped with addictions counseling resources and to be associated with more outpatient buprenorphine prescribers compared to the community ED setting. While all of the residents and physician assistants surveyed also work in surrounding community EDs, just half of the attending physicians interviewed reported that they also have additional appointments in addition to the academic ED where the study took place. This may have biased our study participants in their views on feasibility of initiating treatment with buprenorphine, thus limiting the generalizability of the study results. The survey results may not be representative of the perception of general EPs due to the inclusion of residents, whose external clinical exposure is largely defined by the residency program leadership and the training site. In addition, our reliance on interview-based accounts of practice may result in social acceptability bias, which may have limited participants’ honest description of their perceptions of OUD and buprenorphine.

## CONCLUSION

Our quantitative and qualitative data showed that the majority of ED clinicians neither blame patients with OUD for the difficulty of managing this complex, chronic disease nor consider OUD in and of itself different from other medical conditions. Although they understand the scientific evidence supporting buprenorphine as a long-term treatment option for OUD, they overwhelmingly feel that they do not have adequate training or resources to initiate buprenorphine in the ED. Our qualitative interviews identified a need for institutional response and support, as well as better facilitation of training for waivers and coordination of follow-up after ED-initiation of buprenorphine.

## Figures and Tables

**Table 1:** Interview guide domains and sample questions.

Domains	Sample questions
Perceptions of current ED-based practices to manage patients seeking treatment for OUD	Can you tell me about your experiences working with OUD patients? How do you feel about your current personal practice when treating patients with OUD?
Perceptions of ED-initiated buprenorphine to treat OUD	What are your thoughts on ED clinicians prescribing buprenorphine in the ED? How do you think your colleagues might feel about an ED-based buprenorphine intervention?
Perceived barriers to initiating buprenorphine treatment in the ED	Do you think it would be practical to initiate buprenorphine in the ED? Why or why not? Tell me about your comfort level with initiating buprenorphine in the ED.
Potential solutions to the identified barriers	What would help facilitate you incorporating buprenorphine into your ED practice.

*OUD*, opioid use disorder.

**Table 2:** Demographics of survey respondents.

	n	%
Gender		
Male	48	51.6%
Female	45	48.4%
Role		
Attending	26	28.0%
Resident	41	44.0%
Physician Assistant	26	28.0%
Years of Practice		
0–5 years	55	59.1%
6–10 years	18	19.4%
>10 years	20	21.5%

**Table 3:** Attitudes towards opioid use disorder (OUD) and buprenorphine treatment by years of practice and roles. Eleven discrete, graded responses were possible for each question, with a score of 10 indicating strongly agree and 0 indicating strongly disagree.

Perception of OUD	Median Response (IQR)							

	All clinicians	Years of Practice			Roles			
	
		< 5 years	≥ 5 years	P value	Attg EP	Resident EP	PA	P value
Opioid use disorder (OUD) is different from other chronic diseases (e.g., diabetes, hypertension) because people who use drugs like heroin or illicit opioids are making a choice.	3 (2–6)	2.5 (1–5)	4 (2–7)	<0.01	5 (3–7)	3 (1–4)	2.5 (1–5)	<0.03α
Opioid use disorder is a treatable disease.	8 (7–10)	8 (7–10)	8 (6–10)	0.66	8 (6–10)	8 (7–10)	8 (7–10)	0.85
I find caring for patients with opioid use disorder as satisfying as my other clinical activities.	3 (2–5)	3.5 (2–5)	3 (2–5)	0.84	3 (1–5)	4 (2–5)	3 (2–7)	0.59
Treating opioid use disorders reduces associated health and social costs by more than the cost of the treatment itself.	8 (7–10)	8 (7–10)	9 (7–10)	0.98	9 (7–10)	8 (8–10)	8 (7–10)	0.59
Patients with opioid use disorder are more challenging to take care of than the average patient.	7 (7–0)	7 (7–9)	8 (7–10)	0.01	8 (7–10)	7 (7–9)	8 (7–10)	0.21
Someone who uses drugs is committing a crime and deserves to be punished.	1 (0–3)	1 (0–3)	1 (0–3)	0.63	1 (0–3)	1 (0–3)	1 (0–2)	0.55

Perception of Buprenorphine Treatment								

I think buprenorphine should be administered in the ED for patients requesting treatment for OUD (with referral for outpatient long-term buprenorphine management)?	7 (5–9)	8 (6–10)	6 (3–9)	<0.01	7 (4–9)	9 (7–10)	5 (2–6)	<0.01^βΔ^
Using medications like methadone and buprenorphine for opioid use disorder is simply replacing one addiction with another.	1 (1–4)	1 (0–3)	3 (1–6)	<0.01	2 (1–4)	1 (0–3)	3 (1–6)	<0.01Δ

α statistically significant difference between attending EP and resident EP.

β statistically significant difference between attending EP and PA.

Δ statistically significant difference between resident EP and PA.

*IQR*, interquartile range; *Attg*, attending; *EP*, emergency physician; *PA*, physician assistant.

**Table 4:** Summary response of current practice (A) and preparedness to care for patients with opioid use disorder (OUD) (B) by years of practice and roles. Eleven discrete, graded responses were possible for each question, with a score of 10 indicating very frequently/very prepared and 0 indicating very infrequently/very unprepared.

	Median Response (IQR)							

	All clinicians	Years of Practice			Roles			
	
		< 5 years	≥ 5 years	P value	Attg EP	Resident EP	PA	P value
Current Practice								
See a patient who asks for help with OUD	5 (3–8)	5.5 (2–8)	5 (3–8)	0.87	5.5 (3–7)	5 (5–7)	6 (2–8)	0.70
Refer a patient to OUD treatment	5 (2–6)	3.5 (1–6)	5 (2–7)	0.22	5 (2–7)	4 (2–6)	5 (2–7)	0.44
Prescribe naloxone	2 (1–6)	2.5 (1–6)	2 (1–7)	0.86	3 (0–7)	3 (1–6)	2 (2–7)	0.99
Preparedness								
Screen for OUD	7 (5–8)	6 (5–8)	7 (5–9)	0.10	8 (4–9)	6 (5–8)	7 (5–9)	0.41
Diagnose OUD	7 (6–8)	7 (6–8)	7 (5–8)	0.82	8 (6–8)	7 (6–8)	6 (5–8)	0.50
Provide brief intervention	6 (3–8)	5 (3–7)	7 (4–8)	0.06	5 (4–8)	5 (3–7)	7 (5–8)	<0.01Δ
Refer to OUD treatment	6 (3–8)	7 (4–8)	6 (3–8)	0.48	5 (2–7)	7 (4–8)	7 (3–8)	0.15
Discuss behavioral therapy	3 (2–6)	3 (2–6)	4 (2–6)	0.29	3.5 (2–7)	3 (1–5)	4 (3–6)	0.10
Discuss medication OUD treatment	4 (2–6)	5 (2–6)	3 (2–6)	0.25	4 (2–6)	4 (2–6)	3 (2–6)	0.90
Discuss overdose prevention and naloxone	8 (6–9)	7 (6–9)	8 (7–10)	<0.03	8 (6–10)	7 (5–9)	9 (8–10)	<0.01Δ
Discuss harm reduction	7 (5–8)	7 (5–8)	7 (5–9)	0.12	7.5 (5–9)	6 (5–7)	7.5 (5–9)	<0.02α

α statistically significant difference between attending EP and resident EP.

β statistically significant difference between attending EP and PA.

Δ statistically significant difference between resident EP and PA.

*OUD*, opioid use disorder; *IQR*, interquartile range; *Attg*, attending; *EP*, emergency physician; *PA*, physician assistant.

**Table 5:** Demographics of interviewees.

	n	%
Gender		
Male	11	64.7%
Female	6	35.3%
Fellowship Training		
Completed	8	47.1%
Not completed	9	52.9%
Current Practice Setting		
Academic ED only	8	47.1%
Academic ED and community ED	9	52.9%
Years of Practice		
0–5 years	4	23.5%
6–10 years	2	11.8%
>10 years	11	64.7%
Median 12 (IQR 9–20)		

*IQR*, interquartile range; *ED*, emergency department.

**Table 6:** Clinician-level barriers to emergency department-initiated buprenorphine and potential solutions with supporting quotes.

	Barriers	Solutions
Clinician-level	1. Length of training to prescribe buprenorphine “That’s a little bit ludicrous. I mean, I have much more dangerous drugs that I don’t get 10 hours of training on that I can read about, I can go to a lecture, I can learn about probably – and again, I could be wrong. This could be a very complicated drug, although I don’t think it is. Why are they putting barriers in front of the care providers? You know, be safe. Don’t just say, here, give this medication. People should know about it. But eight hours for one medicine that treats one disorder? That’s a little bit harsh.”	1. Providing training incentives and streamlining process for training, which includes all members of ED team “I think if this is a hospital or institution-wide initiative, I think getting compensated for the time I spend getting the additional training to be able to write for the script, as well as any kind of licensing costs paid for by the hospital would be I think a nice sign or it’s a signal from the hospital of the importance of this issue and the support that they’re willing to give for this.”
	2. Time-consuming nature of building therapeutic relationships and initiating buprenorphine “What’s that like? How long does it take? Is it like a mental health office visit where you sit down and counsel them for 45 minutes? If that’s what’s involved with this stuff, then I can imagine that nobody’s got time for that.”	2. Dedicating staff for identifying patients and initiating buprenorphine in the ED “If you could do all of that, you have like a dedicated – like a SWAT team that came down, like an addiction team, identify this patient, do all that, figure out, is this an appropriate patient to prescribe buprenorphine?”
	3. Lack of immediate impact on patients “... to take time [initiating buprenorphine in the ED]... then the outcome is not immediate. And then my gratification for it is prolonged. That’s why I may not feel as that – it’s not – so, that’s the downside of doing something like this in the emergency department. You don’t see the immediate outcome. And then you’re like, oh, why do I have to do this?”	3. Creating a rapid feedback system to highlight the impact of ED-initiated buprenorphine treatment on patients “People like me, if you ask me to do something and it’s all really evidence based and it’s the great thing to do for the patient but if I don’t get the personal feedback on what I did actually made a difference for that patient, it’s less likely for me to continue doing it even though I know in research papers it’s been efficacious.”

**Table 7:** System-level barriers to emergency department-initiated buprenorphine and potential solutions with supporting quotes.

	Barriers	Solutions
System-level	1. Lack of follow-up mechanism or warm hand-off. “I mean, for what the resources are, I feel like it’s fine. But it’s definitely not sufficient. When we have somebody who’s diabetic and comes in with high blood sugar, either they need to go back to see their PCP or we even have a program where we can get them seen in the endocrine clinic within the next 48 hours. Like, we really – we have things in place to not let those kinds of patients fall through the cracks. But with opioid and substance abuse disorders, there’s all sorts of falling through the cracks.”	1. Ensuring electronic health record integration that include ordering referrals, checking past prescriptions, and sharing individualized care plans. “Like a medical record that we could tap in or [see] patterns of use, not just opiate use but of healthcare system use – if I could see all that, I would feel better, I think. Then I get a better sense of how the patient’s used the healthcare system and how accessible it is to them and how tight the safety net is with them.” “We do have treatment plans for chronic plan that are really effective. We have patient populations, like for example, sickle cell patients with vaso-occlusive crisis. So it could be very much like that [for patients on buprenorphine] – an individualized plan.”
	2. Affordability of buprenorphine and pitfalls in payment models. “Is there an insurance issue? [Patients] could be the ideal candidate, but [if] they don’t have insurance or their insurance doesn’t cover it, [they will be] paying out-of-pocket. Or then they can’t get to the clinic or they can’t get follow-up. Yeah, the healthcare system is tough to navigate sometimes.”	2. Providing ready-to-go buprenorphine supply or in a depot form. “But if we had ready-made, one-week supplies or three-day supplies, I think that would increase the likelihood that patients actually were able to access it and take it appropriately.” “If there was a longer term and non-divertible, like a depo shot for example or something like that, I think that would be ideal just because it – you know that they’re going to receive it. They’re not going to divert it.”
	3. Likely increase in patient volume. “I fear if that word gets out, then we see a 15 percent rise in ED visits for, please give me buprenorphine, which I don’t think we want. I think what we really would like to see is that this becomes a more ubiquitous treatment as an outpatient so we actually see fewer of these patients in overdose in the ED. I worry about the buprenorphine-prescribing workload being shifted to emergency physicians.”	3. Institutionalizing clear protocols for ED-initiation of buprenorphine. “We have pathways for atrial fibrillation, starting blood thinners, and that’s like really well thought out, and most people have no problem with that. I think it would be a similar thing with buprenorphine. But, I think people just need assurance that it’s not unsafe for the patient and for themselves, like medically and legally.”

PCP, primary care physician; ED, emergency department.
